# Conserved genetic basis for microbial colonization of the gut

**DOI:** 10.1016/j.cell.2025.04.018

**Published:** 2025-04-25

**Authors:** Menghan Liu, Sydney B. Blattman, Mai Takahashi, Nandan Mandayam, Wenyan Jiang, Panos Oikonomou, Sohail F. Tavazoie, Saeed Tavazoie

In the originally published version of this article, we noted two errors: for Figure 3D, although the results and methods correctly state “streptomycin,” the figure graphic was mistakenly labeled as “spectinomycin”; for Figure 4C, the graphic showed mismatched promoters and gene names. Both figures have been corrected in the original article and below. These corrections do not affect any of the results or conclusions of this paper. The authors apologize for any inconvenience due to the errors.

## Figures and Tables

**Figure 3D.**
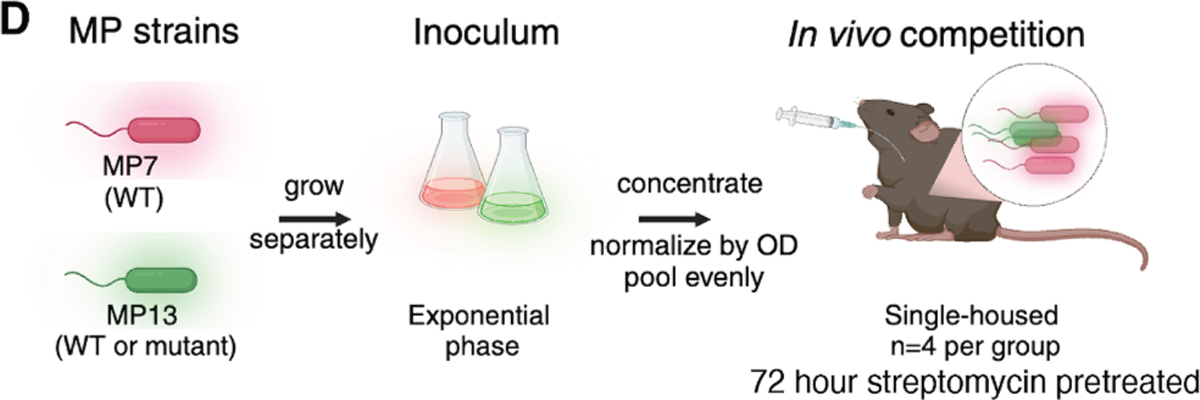
YigZ and TrhP are required for *E. coli* MP13 colonization (corrected)

**Figure 3D.**
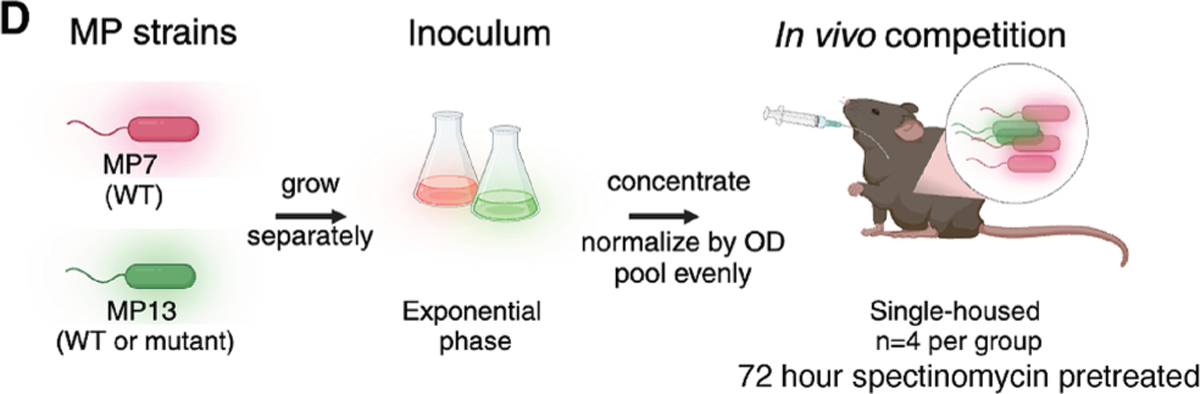
YigZ and TrhP are required for *E. coli* MP13 colonization (original)

**Figure 4C.**
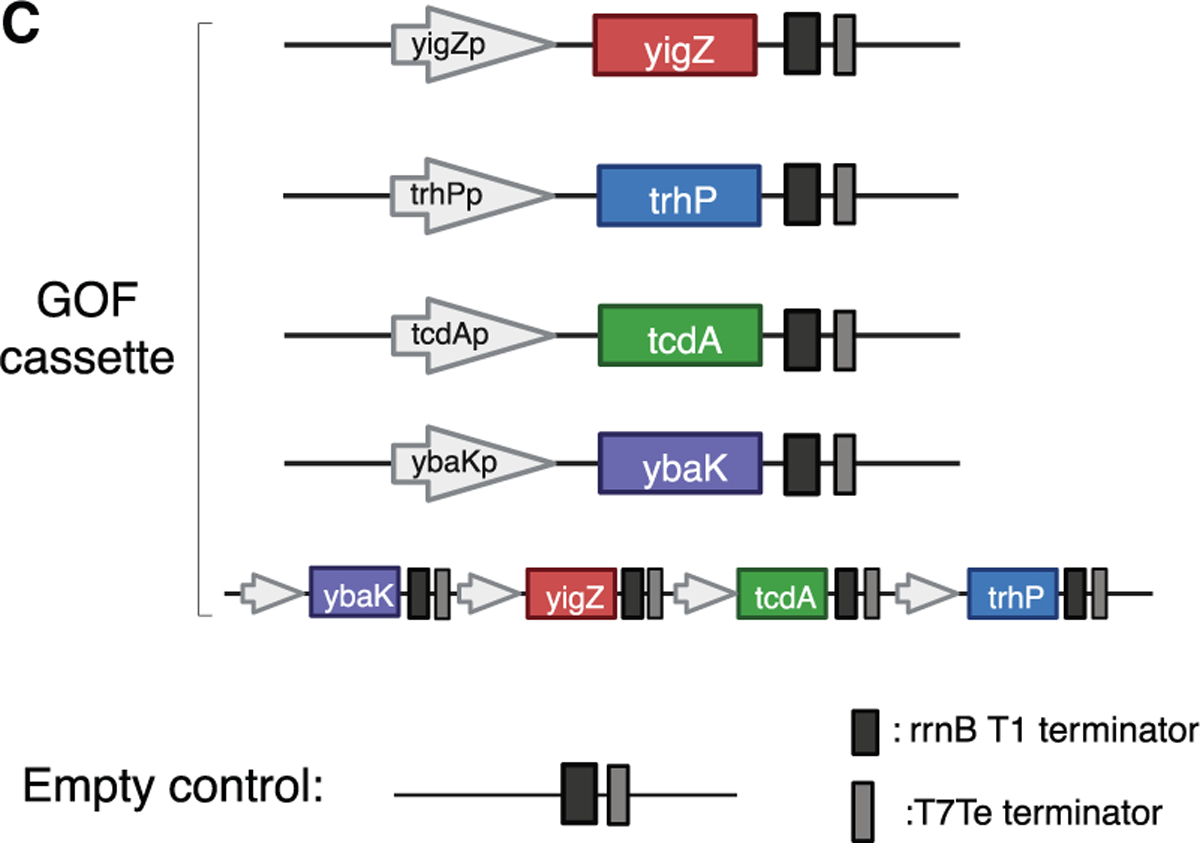
YigZ overexpression is sufficient to enhance gut colonization of *E. coli* MG1655 (corrected)

**Figure 4C.**
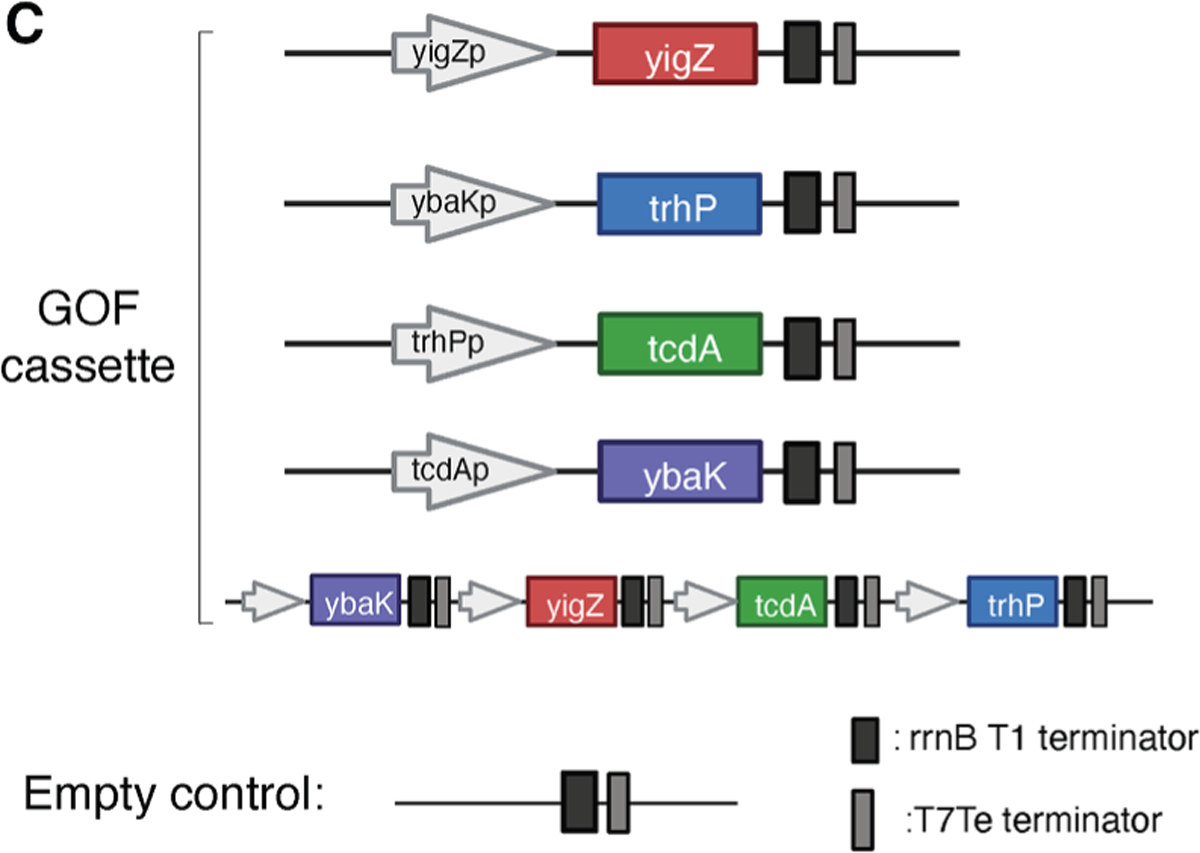
YigZ overexpression is sufficient to enhance gut colonization of *E. coli* MG1655 (original)

